# Protocol for Mouse Embryonic Aorta–Gonad–Mesonephros (AGM) Region Frozen Sectioning and Immunofluorescence

**DOI:** 10.21769/BioProtoc.5828

**Published:** 2026-09-20

**Authors:** Yan Jia, Sixuan Huo, Mei Li

**Affiliations:** School of Life Science and Technology, Shandong Second Medical University, Weifang, China

**Keywords:** Aorta–gonad–mesonephros, Hematopoietic stem cells, Endothelial-to-hematopoietic transition, Cryosectioning, Immunofluorescence

## Abstract

The aorta–gonad–mesonephros (AGM) region is the site where hematopoietic stem cells (HSCs) first emerge during development, and is therefore widely used to study in vivo hematopoiesis and to discover novel regulatory mechanisms. The endothelial-to-hematopoietic transition (EHT) process can be directly observed via immunofluorescence on frozen sections of the AGM region. However, the mouse AGM region is extremely delicate and lies deep within the embryo, between the notochord and the somatic mesoderm. Here, we present a step-by-step protocol covering embryo collection, fixation, dehydration, and embedding with a defined orientation, followed by frozen sectioning, immunofluorescence staining, and confocal imaging. The protocol is highly reproducible and easy to follow and provides clear instructions on orienting the embryo and anatomically locating AGM. By filling a technical gap, the protocol can enable researchers to reliably study HSC emergence and EHT in the mouse embryonic AGM.

Key features

• Provides detailed instructions on embryo embedding orientation and microscopic observation during cryosectioning to precisely locate the mouse AGM region.

• Covers all steps from embryo harvesting, cryosectioning, and immunofluorescence to confocal imaging, ensuring high reproducibility.

• Developed for delicate mouse embryonic AGM tissue (e.g., E9.5–E11.5), enabling direct observation of EHT via immunofluorescence.

## Graphical overview


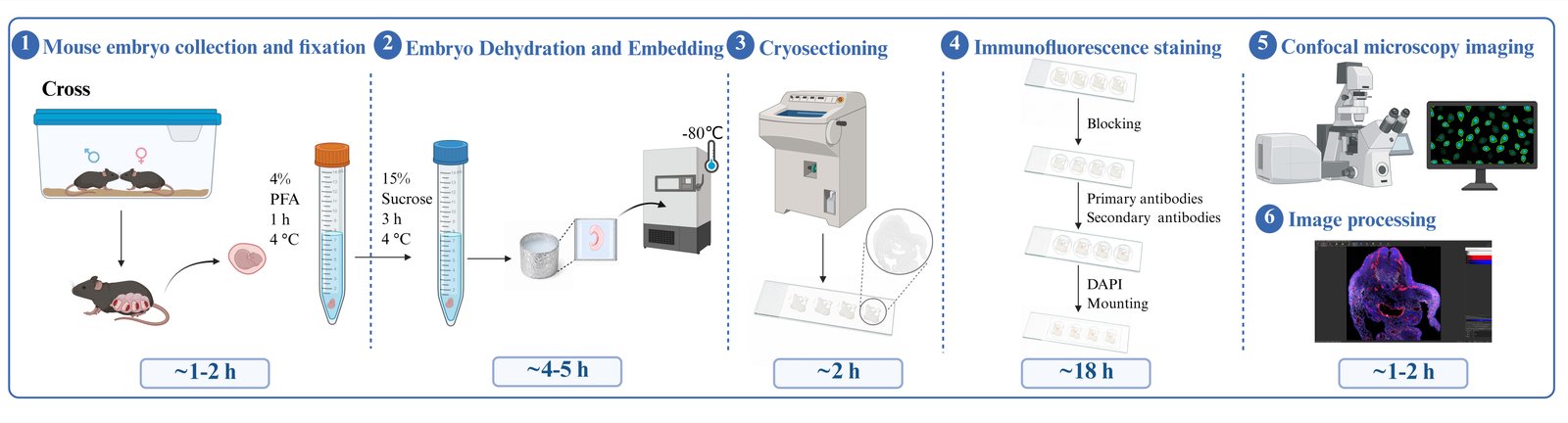


**Graphical overview of immunofluorescence staining and imaging of E10.5 mouse aorta–gonad–mesonephros (AGM) cryosections**

## Background

Hematopoietic stem cells (HSCs) reside at the apex of the hematopoietic hierarchy, possessing the capacity for self-renewal and differentiation into all downstream blood cell lineages [1–3]. HSCs are generated during definitive hematopoiesis in the embryonic stage in mice, arising from a specialized population of hemogenic endothelial cells (HECs) in the aorta–gonad–mesonephros (AGM) region through endothelial-to-hematopoietic transition (EHT) [4,5]. During this process, HECs give rise to immature hematopoietic cells, including pre-HSCs and hematopoietic progenitors. These cells subsequently form intra-aortic hematopoietic clusters (IAHCs) attached to the aorta [4,6]. Elucidating the cellular and molecular mechanisms underlying HSC origination in the AGM region is of great significance for understanding normal hematopoietic development and for guiding the induction of HSCs from pluripotent stem cells in vitro [7,8].

Previous studies on the AGM region have systematically revealed the dynamic stages of hematopoietic development and key molecular markers, in which immunofluorescence has played an indispensable role. For example, this technique enables the identification of intermediate states of hematopoietic development, such as the discovery and in situ localization of VE-cadherin^+^CD45^+^ type II pre-HSCs [9]. By co-staining with the hematopoietic transcription factor RUNX1 and the endothelial markers CD31 or VE-Cadherin, immunofluorescence further allows the characterization of various hematopoietic molecules in the aorta. For instance, angiotensin-converting enzyme (ACE) was identified as a marker of HECs, while CD44 serves as a marker of EHT [10,11]. Also, immunofluorescence can be used to validate the specific expression pattern of newly generated fluorescent reporter mice (e.g., *Gfi1:H2B-Tomato* and *Runx1+23GFP*) in the AGM region, and to morphologically determine the intermediate state of the transgenic-labeled hematopoietic precursors [12,13]. Moreover, this technique has been applied to assess the effects of genetic perturbations on hematopoietic development. For example, after endothelial-specific deletion of *Meis1, Meis1* ^flox/flox^;*VEC-Cre;Runx1+23GFP* embryos at embryonic day (E) 10.5 were analyzed by GFP and CD31 expression in the dorsal aorta, and the number of IAHCs was quantified to evaluate hematopoietic defects [14]. In summary, frozen-section immunofluorescence provides clear and single-cell resolution visualization for studying HSC emergence in the AGM region. It complements flow cytometry, in vitro functional assays, and single-cell transcriptomic sequencing, and thus possesses irreplaceable value in this field.

Although frozen-section immunofluorescence of the AGM region has been used in numerous studies, and its basic procedures have been described, critical details for precisely locating the AGM region—such as the optimal embryo orientation for embedding and how to identify AGM-containing sections during cryosectioning—have not been thoroughly addressed. The lack of such details makes it difficult for beginners to consistently obtain the required sections, leading to relatively poor experimental reproducibility. This protocol provides, for the first time, systematic guidance on embryo collection, embedding, anatomical landmark recognition during cryosectioning, and confocal imaging. Therefore, it can effectively improve section consistency and reproducibility, reduce waste of embryos and time, and offer a reliable methodological foundation to advance AGM-related hematopoietic research.

## Materials and reagents

**Biological materials**

1. E9.5, E10.5, and E11.5 wild-type C57BL/6 mouse embryos obtained from timed matings (bred in-house)

2. E10.5 C57BL/6 *Cdh5-Cre; Rosa26-CAG-loxP-STOP-loxP-tdTomato* reporter mouse embryos obtained from timed matings of mice bred in-house (*Cdh5-Cre* mice were purchased from Cyagen Biosciences; *Rosa26-CAG-loxP-STOP-loxP-tdTomato* mice were generated by Cyagen Biosciences; used in Figure S2)

**Reagents**

1. α-minimum essential medium (α-MEM) (Cytiva, catalog number: SH30265.01)

2. Fetal bovine serum (FBS) (Thermo Fisher Scientific, Gibco, catalog number: A5256701)

3. Phosphate buffered saline (PBS), 10× (M&C Gene Technology, catalog number: CC008.1)

4. 4% paraformaldehyde (PFA) (Servicebio, catalog number: G1101)

5. Sucrose (Sinopharm, catalog number: 10021418)

6. Triton X-100 (Diamond, catalog number: A110694)

7. 75% ethanol (Sinopharm Chemical Reagent, catalog number: 80176965)

8. DAPI (Beyotime, catalog number: C1002)

9. Anti-fade mounting medium (Invitrogen, catalog number: P36984)

10. Tissue-Tek O.C.T. compound (Sakura Finetek, catalog number: 4583)

11. Rat anti-CD31/PECAM-1 primary antibody (Santa Cruz, catalog number: sc-18916)

12. Mouse anti-RUNX1 primary antibody (Santa Cruz, catalog number: sc-365644)

13. Alexa Fluor 488-conjugated goat anti-mouse secondary antibody (Invitrogen, catalog number: A28175)

14. Alexa Fluor 546-conjugated goat anti-rat secondary antibody (Invitrogen, catalog number: A11081)

**Solutions**

1. 10% FBS/α-MEM (see Recipes)

2. 10% FBS/PBS (see Recipes)

3. 15% sucrose/PBS (see Recipes)

4. Immunofluorescence blocking buffer (blocking buffer) (see Recipes)

5. Antibody dilution buffer (see Recipes)

**Recipes**

**1. 10% FBS/α-MEM**

**Table:** 

Reagent	Final concentration	Volume
α-MEM	N/A	45 mL
FBS	10%	5 mL
Total	N/A	50 mL

Thaw FBS at 4 °C overnight. Prepare 45 mL of α-MEM in a sterile 50 mL conical tube. Add 5 mL of FBS to the α-MEM and mix gently by inversion. Avoid vigorous shaking to prevent foaming. The 10% FBS/α-MEM solution can be stored at 4 °C for up to one week.

*Note: Heat inactivation of FBS is not necessary.*

**Caution:** Freeze FBS in aliquots to avoid freeze/thaw cycles and store the aliquots at -20 °C.

**2. 10% FBS/PBS**

**Table:** 

Reagent	Final concentration	Volume
FBS	10%	5 mL
10× PBS	1×	5 mL
Autoclaved distilled water	N/A	40 mL
Total	N/A	50 mL

Thaw FBS at 4 °C overnight. Prepare 40 mL of autoclaved distilled water in a sterile 50 mL conical tube. Add 5 mL of 10× PBS and mix thoroughly. Add 5 mL of FBS to the PBS and mix gently by inversion until homogeneous. The 10% FBS/PBS solution can be stored at 4 °C for up to one week.

**3. 15% sucrose/PBS**

**Table:** 

Reagent	Final concentration	Quantity
Sucrose	15% w/v	7.5 g
10× PBS	1×	5 mL
Autoclaved distilled water	N/A	Up to 50 mL
Total	N/A	50 mL

Prepare approximately 35 mL of autoclaved distilled water in a sterile 50 mL conical tube. Add 5 mL of 10× PBS and mix thoroughly. Weigh 7.5 g of sucrose and add it to the tube. Mix gently at room temperature until the sucrose is completely dissolved. Adjust the final volume to 50 mL with autoclaved distilled water and mix well. The 15% sucrose/PBS can be stored at 4 °C for up to one week.

**4. Blocking buffer**

**Table:** 

Reagent	Final concentration	Volume
10× PBS	1×	10 mL
FBS	10%	10 mL
Triton X-100	0.2%	0.2 mL
Autoclaved distilled water	N/A	79.8 mL
Total	N/A	100 mL

Prepare 79.8 mL of autoclaved distilled water in a sterile container. Add 10 mL of 10× PBS and mix thoroughly. Add 10 mL of FBS and mix gently until homogeneous. Add 0.2 mL of Triton X-100 and mix slowly to avoid foaming. The blocking buffer is recommended to be prepared fresh and used immediately.

**5. Antibody dilution buffer**

**Table:** 

Reagent	Final concentration	Volume
10× PBS	1×	10 mL
FBS	3%	3 mL
Triton X-100	0.2%	0.2 mL
Autoclaved distilled water	N/A	86.8 mL
Total	N/A	100 mL

Prepare 86.8 mL of autoclaved distilled water in a sterile container. Add 10 mL of 10× PBS and mix thoroughly. Add 3 mL of FBS and mix gently until homogeneous. Add 0.2 mL of Triton X-100 and mix slowly to avoid foaming. The antibody dilution buffer should be prepared fresh and used immediately.

**Laboratory supplies**

1. Sterile 50 mL conical tubes (CellPro, catalog number: 801501)

2. Sterile 15 mL conical tubes (CellPro, catalog number: 801151)

3. Sterile 35 mm cell culture dishes (Thermo Fisher Scientific, catalog number: 150460)

4. 60 mm glass culture dish (Changde BKMAM, catalog number: 120403007)

5. Adhesion microscope slides (Citotest, catalog number: 188105)

6. Coverslips (Citotest, catalog number: 10212450C)

7. Humidified chamber (Sangon Biotech, catalog number: E678020)

8. Light-protective staining box (Nantong Supin, for 24 slides)

9. Hydrophobic barrier pen (Daido Sangyo, catalog number: 0010G)

10. Lint-free wipes (Kimtech, catalog number: 34155)

11. Aluminum foil (Labshark, catalog number: 130410222)

12. Aluminum foil molds (in-house)

13. 31-gauge (G) insulin syringe (Shanghai Kindly, catalog number: U-40)

14. Iris forceps (Sangon Biotech, catalog number: F519023)

15. Iris scissors (Sangon Biotech, catalog number: F519232)

16. Size 5 fine forceps (Fine Science Tools, catalog number: 11254-20)

17. Cryostat specimen chucks (Leica Biosystems, catalog number: 14037008587)

18. Cryostat blades (Leica Biosystems, catalog number: 14035838925)

19. Micropipettes, 0.5–10, 20–200, and 100–1,000 μL (Eppendorf, catalog numbers: 3123000020, 3123000055, and 3123000063)

20. Pipette tips, 0.1–10, 2–200, and 50–1,000 μL (Biosharp, catalog numbers: BS-10-T, BS-200-T, and BS-1000-T)

21. Sterile disposable serological pipets, 5, 10, and 25 mL (Corning, catalog numbers: CLS4487, CLS4488, and CLS4489)

22. Pipette controller, 0.1–100 mL (Eppendorf, catalog number: 4430000018)

23. Glass graduated cylinders, 50 and 100 mL (SolelyBio, catalog numbers: SBM0085 and SBM0233)

24. Glass beakers, 100 and 250 mL (SolelyBio, catalog numbers: SBM0084 and SBM0382)

25. Glass reagent bottles with screw caps, 250, 500, and 1,000 mL (Shuniu, catalog numbers: SN03250, SN03500, and SN031000)

## Equipment

1. Stereomicroscope (Nikon, model: SMZ745T)

2. Cryostat (Leica Biosystems, model: CM1860)

3. Confocal laser scanning microscopes (Zeiss, model: LSM900)

4. -80 °C freezer (Haier, model: DW-86L486)

5. 4 °C refrigerator (Haier, model: BCD-520WDPD)

6. 37 °C incubator (Shanghai Zhicheng, model: ZXGP-B2080)

7. Orbital shaker (Changzhou Nuoji, model: KTS-103H)

8. Liquid nitrogen storage container (Chengdu Jinfeng, model: YDS-175-216)

9. Carbon dioxide (CO_2_) euthanasia chamber (Braintree Scientific, model: COIC)

## Software and datasets

1. ZEN (blue edition) microscopy software (Zeiss, version: 3.6)

2. Imaris x64 (Oxford Instruments/Bitplane, version: 9.0.1)

## Procedure

**A. Mouse embryo collection and fixation**

1. Set up timed mating by housing one C57BL/6 male mouse with two female mice in one cage between 16:00 and 17:00.

2. Check female mice for the presence of a vaginal plug between 8:00 and 9:00 the following morning.

3. Designate noon of the day when a vaginal plug is detected as E0.5.

4. Separate females with vaginal plugs immediately and house them individually.

**Critical:** Accurate determination of embryonic day is essential for obtaining embryos at the correct developmental stage.

5. Maintain pregnant females in a stable and quiet environment with minimal disturbance until E10.5.

6. Euthanize pregnant females at E10.5 with CO_2_, followed by cervical dislocation, according to the approved institutional animal care and use protocol. Confirm death before proceeding with embryo collection.

7. Immerse the abdominal area of the euthanized mouse in 75% ethanol for 1–2 min for surface sterilization.

8. Using sterile forceps, gently lift the abdominal skin and make a small incision with sterile scissors. Extend the skin incision to expose the abdominal wall, then carefully lift and cut the peritoneum to expose the abdominal cavity (Figure 1A).

9. Carefully collect the uterine horns containing the embryos from both sides of the abdominal cavity using sterile forceps and scissors. Transfer them into a glass culture dish containing 4 °C prechilled 10% FBS/α-MEM (Figure 1B). Place the culture dish on ice during embryo dissection to help maintain cell viability.

10. Under a stereomicroscope, isolate individual embryos from the uterus in the cell culture dish. Using size-5 fine forceps, carefully tear open the uterine wall, remove the endometrial and placental tissues, and release each embryo together with the intact yolk sac (Figure 1C, D).

11. Peel away the yolk sac to expose the embryo (Figure 1E, F). Count somites under the stereomicroscope to confirm the developmental stage.

**Critical:** Determine embryo stage using both plug timing and somite number to reduce developmental variation. Embryos at E10.5 are typically identified as having 36–40 somite pairs.

12. Transfer embryos into a 15 mL conical tube containing 5 mL of 4% PFA and leave them undisturbed at 4 °C for 1 h.

**Critical:** Fixation time is critical for successful co-staining of transcription factors and CD31. For E10.5 embryos, the fixation time should be maintained at 1 h. The same fixation condition is also applicable to E9.5 and E11.5 embryos.

13. Remove the PFA and wash embryos twice with cold PBS for 5 min each wash.

**Figure 1.**
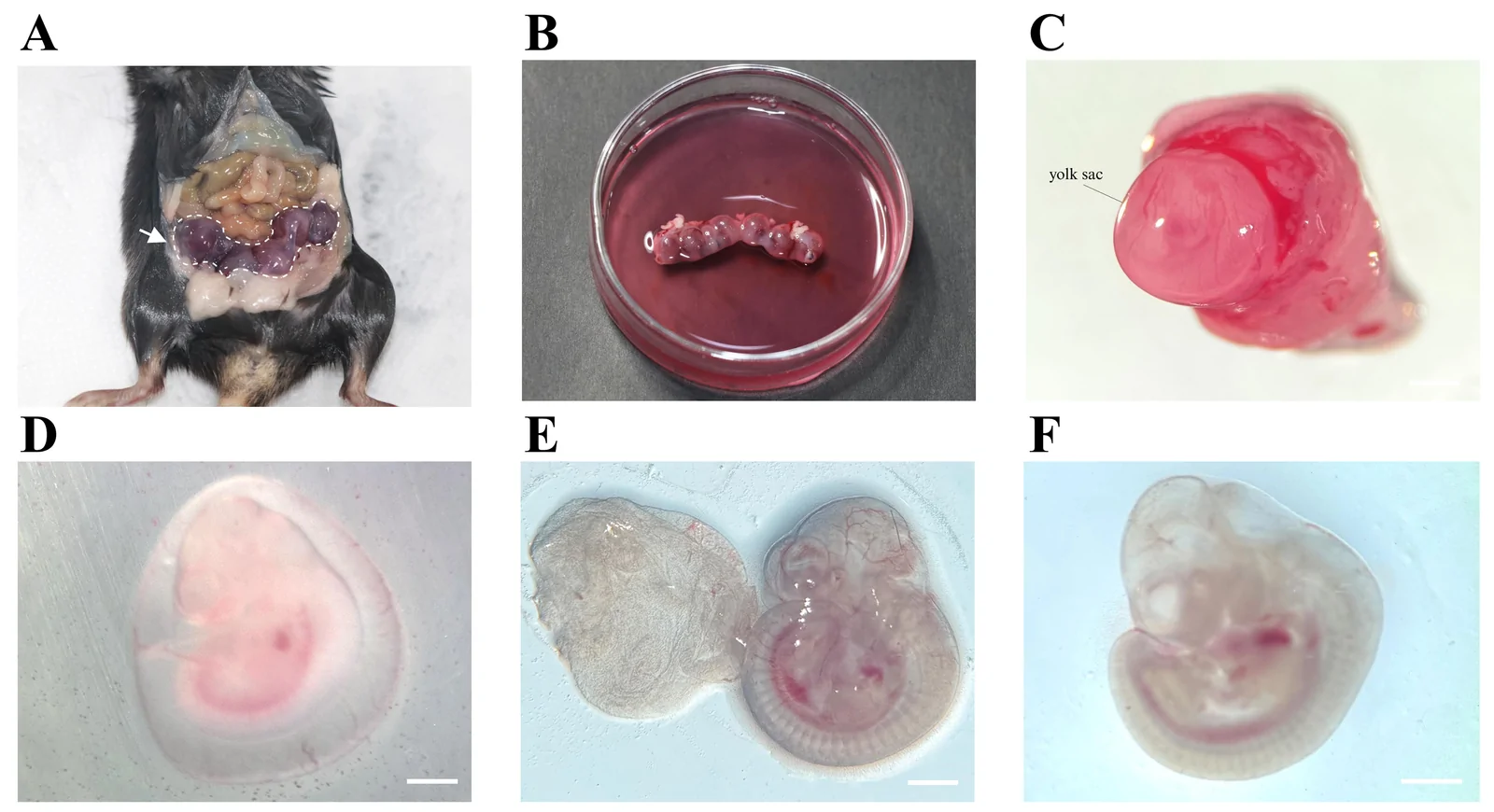
Representative images of E10.5 mouse embryo dissection. (A) E10.5 uterine horns exposed in the abdominal cavity after opening the abdominal skin and peritoneum. The uterine horns are outlined with white dashed lines and indicated by a white arrowhead. (B) Uterine horns containing embryos maintained in prechilled 10% FBS/α-MEM. (C) The uterine wall and decidual tissue are opened to expose the intact yolk sac surrounding the embryo. (D) An intact E10.5 mouse embryo fully encased in the yolk sac. (E) The yolk sac is opened and positioned beside the embryo, exposing the embryo before complete yolk sac removal. (F) E10.5 mouse embryo after removal of the yolk sac. The developmental stage is determined by somite count (36–40 somites). Scale bars: 1 mm.

**B. Embryo dehydration and embedding**

1. Transfer the fixed embryos into 15% sucrose/PBS and leave them undisturbed at 4 °C for 3 h or until they sink to the bottom of the tube.

**Critical:** Dehydration times longer than 4 h cause excessive dehydration, while times shorter than 2 h result in insufficient cryoprotection. The same dehydration condition is also applicable to E9.5 and E11.5 embryos.

2. Prepare cylindrical embedding molds approximately 1.5 cm in diameter and 1.2 cm in height using aluminum foil (Figure 2A).

3. Add Tissue-Tek O.C.T. compound to each mold, filling it to approximately two-thirds of its height.

4. Under a stereomicroscope, trim the embryo by removing the regions above the forelimbs and below the hindlimbs, as well as visceral organs (Figure 2B, C).

5. Transfer the trimmed embryo to the top surface of a cell culture dish lid and carefully remove excess surrounding liquid.

6. Cover the embryo with a small drop of O.C.T. compound to ensure it is fully enveloped.

7. Transfer the trimmed embryo into the embedding mold containing O.C.T. compound using forceps.

8. Orient the embryo vertically in the O.C.T. using a 31 G insulin needle. Gently adjust its position until the anterior end is facing upward and the posterior end downward, keeping the embryo centered in the mold (Figure 2D).

**Critical:** Correct embryo orientation is essential for obtaining consistent transverse sections through the AGM region.

9. Remove visible air bubbles around the embryo using a 31 G insulin needle.

10. Rapidly freeze the mold by placing it directly into liquid nitrogen until the O.C.T. compound solidifies completely.

11. Store frozen embryo blocks at -80 °C until cryosectioning.

**Pause point:** Frozen blocks can be stored at -80 °C for up to 6 months.

**Figure 2.**
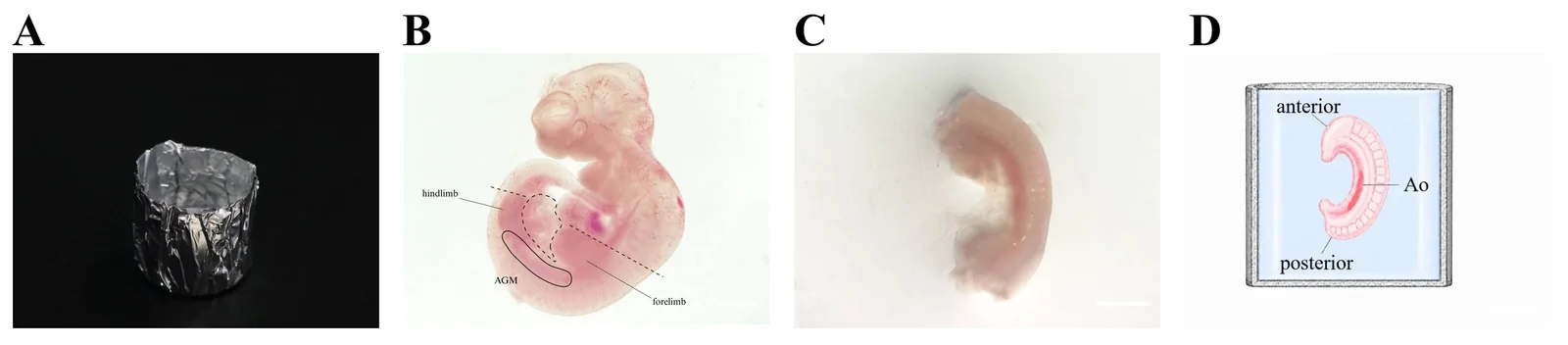
Embryo trimming and orientation for O.C.T. embedding. (A) Image illustrating an in-house aluminum foil mold prepared for O.C.T. embedding. (B) Lateral view of an E10.5 mouse embryo. Two straight black dashed lines mark the trimming planes above the forelimbs and below the hindlimbs. The visceral organs to be removed are outlined with a black dashed line, and the aorta–gonad–mesonephros (AGM) region is outlined with a black solid line. (C) Representative image of an E10.5 mouse embryo after removal of the regions anterior to the forelimbs and posterior to the hindlimbs and visceral organs. (D) Schematic diagram illustrating the embryo orientation used for embedding, with the anterior end positioned upward and centered in O.C.T. compound. Ao: aorta. Scale bar: 1 mm.

**C. Cryosectioning**

1. Set the cryostat chamber temperature to -20 °C. The cryostat chamber should be maintained at -20 °C throughout the following steps.

2. Mount the frozen O.C.T.-embedded embryo block onto the specimen chuck with the anterior end facing the specimen chuck by placing a small amount of fresh O.C.T. compound between them. Then, freeze the assembly in the rapid cooling zone of the cryostat.

3. Position the specimen chuck on the orientable specimen head (Figure 3A).

4. Trim the block at a thickness of 60 μm until embryo tissue becomes visible (Figure 3B).

5. Adjust the section thickness to 10 μm and collect cryosections onto adhesion microscope slides.

*Note: For a correctly oriented E10.5 embryo, the AGM region is typically reached after collecting approximately 60 consecutive 10-μm sections from the point at which embryonic tissue first becomes visible. This number may vary depending on embryo size and embedding orientation.*

6. Observe cryosections under a light microscope and identify the AGM region using anatomical landmarks, including the neural tube, aorta, and mesonephric tubules (Figure 3C). A representative image of a section that lacks correct AGM anatomy is shown in Figure S1.

**Critical:** Accurate identification of the AGM region based on its anatomical position is essential for subsequent immunofluorescence.

7. Store slides at -80 °C or proceed directly to immunofluorescence staining.

**Pause point:** Cryosections can be stored at -80 °C before staining. Avoid repeated freeze/thaw cycles.

**Figure 3.**
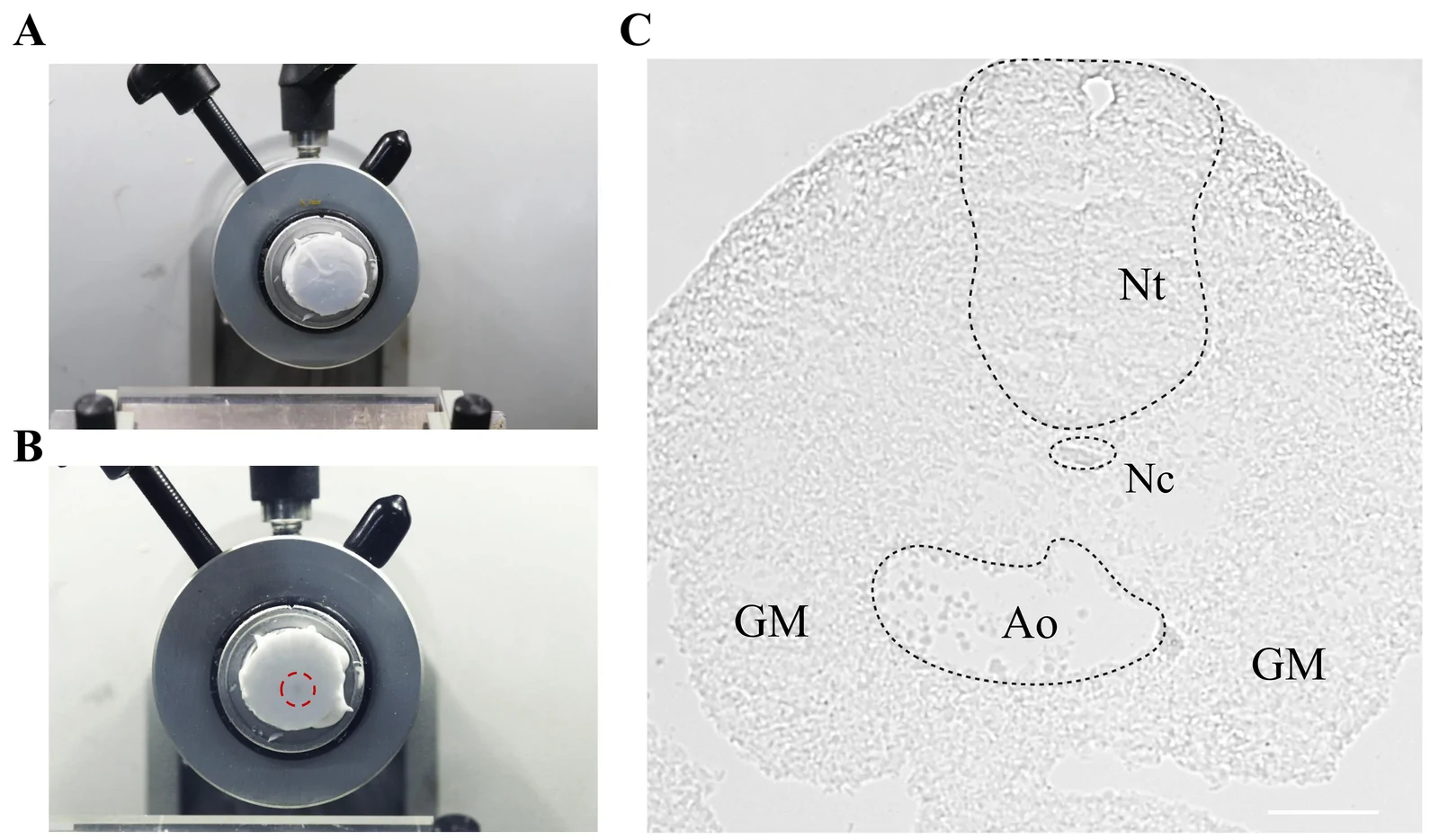
Anatomical identification of the aorta–gonad–mesonephros (AGM) region in E10.5 mouse embryo cryosections. (A) Embedded embryo block on the cryostat chuck, with the anterior end of the embryo facing inward. (B) After trimming, the embryo tissue becomes visible within the red circle. (C) Brightfield image of an E10.5 mouse embryo transverse cryosection showing the major anatomical landmarks of the AGM region. The aorta is positioned centrally beneath the neural tube and is flanked by the gonads and mesonephros. Nt, neural tube; Ao, aorta; G, gonad; M, mesonephros; Nc, notochord. Scale bar: 50 μm.

**D. Immunofluorescence staining**

1. Retrieve the frozen sections from the –80 °C freezer and thaw them in a 37 °C incubator for 30 min.

*Note: Include only sections with intact AGM morphology. Exclude sections with tissue detachment, severe folding, or tissue cracking.*

2. Place slides in a light-protective staining box.

3. Gently wash slides three times with PBS, 5 min per wash, on a shaker set to 20 rpm.

*Note: Do not use excessive shaking speed, as embryonic cryosections detach easily from the slide.*

4. Remove excess PBS and gently wipe areas outside the tissue using lint-free wipes.

5. Draw a hydrophobic barrier around each tissue section using a hydrophobic pen.

6. Add 30–50 μL of blocking buffer to each section and incubate at room temperature for 45 min in a humidified chamber (Figure 4A, B).

*Note: If a commercial humidified chamber is unavailable, a suitable chamber can be prepared using a sealable plastic box. Add a shallow layer of distilled water to the bottom of the box and place the slides horizontally on a raised slide rack above the water. Ensure that the slides do not come into direct contact with the water. Close the lid during incubation to maintain a humid environment and minimize evaporation of the antibody solution.*

7. Remove the blocking buffer by gently shaking off the slide.

8. Add 40 μL of diluted primary antibodies in antibody dilution buffer to each section and incubate at 4 °C overnight in a light-protective humidified chamber with water added to the bottom (Table 1).

**Critical:** Keep sections covered with solution throughout staining. Drying of the tissue sections will increase background staining.

**Table 1.** Primary antibodies used for immunofluorescence staining

Primary antibody	Host species	Clone	Dilution	Manufacturer	Catalog number
CD31/PECAM-1	Rat	MEC 13.3	1:200	Santa Cruz	sc-18916
RUNX1	Mouse	A-2	1:200	Santa Cruz	sc-365644

9. Wash slides three times with PBS, 10 min per wash, on a shaker at 20 rpm in the staining box.

10. Add 30 μL of diluted secondary antibodies in antibody dilution buffer to each section and incubate in a humidified chamber at room temperature for 1 h (Table 2).

**Critical:** Protect slides from light after secondary antibody incubation.

**Table 2.** Secondary antibodies used for immunofluorescence staining

Secondary antibody	Target species	Fluorophore	Dilution	Manufacturer	Catalog number
Goat anti-rat IgG	Rat	Alexa Fluor 546	1:1,000	Invitrogen	A11081
Goat anti-mouse IgG	Mouse	Alexa Fluor 488	1:1,000	Invitrogen	A28175

11. Wash slides twice with PBS in the staining box for 10 min each wash on a shaker at 20 rpm.

12. Dilute DAPI at 1:5,000 in antibody dilution buffer and add 30 μL to each section.

13. Incubate slides in a humidified chamber at room temperature for 10 min.

14. Wash slides twice with PBS in the staining box for 10 min each wash on a shaker at 20 rpm.

15. Remove excess liquid and gently wipe areas around the tissue.

16. Add 10 μL anti-fade mounting medium to each section.

17. Place a coverslip gently over the section and avoid air bubbles (Figure 4C, D).

18. Seal the edges of the coverslip with clear nail polish.

19. Store slides at 4 °C in the dark before imaging.

**Critical:** For optimal imaging results, perform confocal imaging within 24 h.

**Figure 4.**
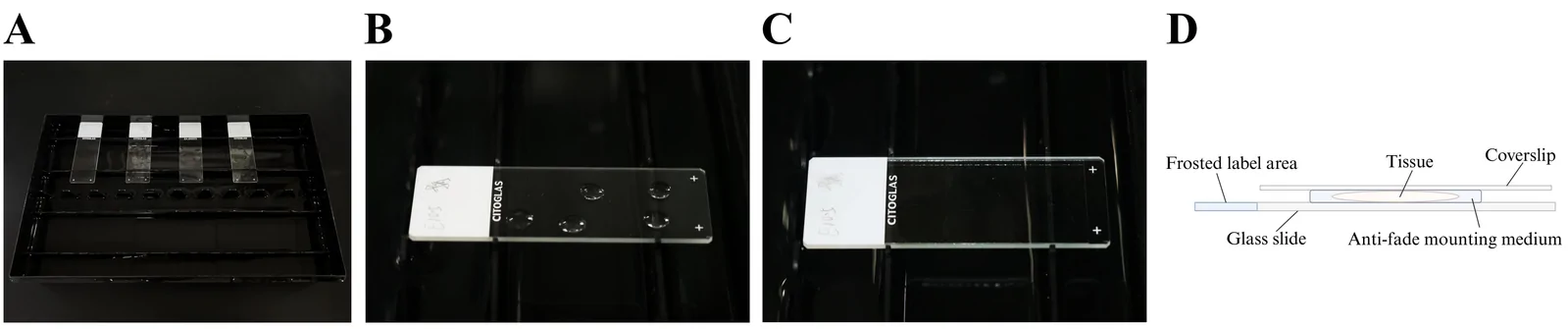
Immunofluorescence staining and mounting of cryosections. (A) Representative image of the humidified chamber used for blocking and antibody incubation, showing the slides positioned inside the chamber and distilled water added to the bottom of the container. (B) Image illustrating the hydrophobic barrier drawn around tissue sections, with the barrier-enclosed area containing blocking solution, primary antibodies, or secondary antibodies. (C) Representative image showing mounting of the stained sections with anti-fade mounting medium. (D) Schematic illustration of the mounted slide, showing the position of the glass slide, tissue section, anti-fade mounting medium, and coverslip.

**E. Confocal microscopy imaging**

1. Acquire fluorescence images using a Zeiss LSM900 confocal microscope with ZEN software.

2. On the microscope, use the DAPI channel with a 20× objective to locate the AGM region based on anatomical landmarks, including the neural tube, aorta, and adjacent mesonephric tubules and mesonephros. The 20× objective allows complete visualization of the entire aorta. Switch to a 40× objective for detailed imaging if required.

**Critical:** Use the DAPI channel for initial localization, as the neural tube is readily identifiable in this channel and helps to confirm the AGM position.

3. Configure fluorescence channels based on the excitation and emission spectra of the fluorophores. For example, DAPI (excitation 405 nm, detection 400–605 nm), CD31-Alexa Fluor 546 (excitation 561 nm, detection 540–620 nm), and RUNX1-Alexa Fluor 488 (excitation 488 nm, detection 450–555 nm). To minimize fluorescence crosstalk caused by spectral overlap, images were acquired using sequential scanning mode, with each fluorophore excited and detected separately.

**Optional:** For higher-resolution imaging, acquire images using Airyscan mode following the manufacturer’s recommended acquisition settings.

4. Optimize laser power and detector gain based on signal intensity, ensuring that the target signal is clearly distinguishable from the background, while avoiding fluorescence overexposure and photobleaching.

**Critical:** Fluorescence overexposure cannot be corrected by subsequent software adjustments and will compromise the quality of the resulting data.

5. For three-dimensional imaging, acquire Z-stack images covering the full thickness of the tissue section. Set the Z-step interval, typically 0.5–1 μm, to fully capture cellular details. A summary of the imaging parameters is provided in Table 3.

6. Save raw images in .czi format for downstream image processing and analysis.

**Table 3.** Confocal imaging parameters for E10.5 mouse aorta–gonad–mesonephros (AGM) immunofluorescence sections

Category	Parameter	Setting
Microscope	Manufacturer and model	Zeiss LSM900
Acquisition mode	Frame size	1,024 × 1,024 pixels
	Scan speed	5
	Direction	Unidirectional
	Zoom factor	0.45
	Averaging	None
	Bits per pixel	16 bits
Channel configuration	Tracks	CD31: Alexa Fluor 546; RUNX1: Alexa Fluor 488, DAPI
	Laser power	Adjust according to signal intensity and avoid photobleaching
	Master gain	Adjust according to signal intensity and avoid overexposure
Scan mode	Sequential scan	Alexa Fluor 546→Alexa Fluor 488→DAPI
Z-stack	Interval	0.5–1 μm
	Range	Cover the whole aorta
Accessories	Objective	20× (NA 0.8, Air)
	Airyscan (optional)	Airyscan 2
File output	Raw data	.czi format

## Data analysis

**A. Image import**

1. Process confocal images using Imaris x64 9.0.1 or higher version. Confirm that the pixel size and Z-step information are properly recognized before analysis.

2. After opening Imaris, switch to the Arena view. Create an assay, drag the acquired Zeiss .czi files into the assay, then create subfolders as needed for subsequent image processing.

*Note: For different experimental groups, create separate groups inside the assay to organize the images.*

**B. Image processing and analysis**

1. In 3D view mode, assign distinct pseudocolors to each image channel. For example, set DAPI to dark blue, CD31 to red, and RUNX1 to white.

2. Adjust brightness and contrast exclusively for visualization.

3. For qualitative analysis, evaluate the number of CD31^+^/RUNX1^+^ double-positive hematopoietic cells (including those embedded in the vessel wall, undergoing budding, and having completed budding) in the aorta. A marked reduction in the number of double-positive cells suggests impaired definitive hematopoiesis [14].

*Note: To ensure reproducibility, at least three embryos per group should be analyzed, with multiple sections per embryo.*

**C. Image export**

Export images of each individual fluorescence channel as well as their merged overlays (with embedded scale bars) in TIFF format for figure preparation. Representative images are shown in Figure 5.

**Figure 5.**
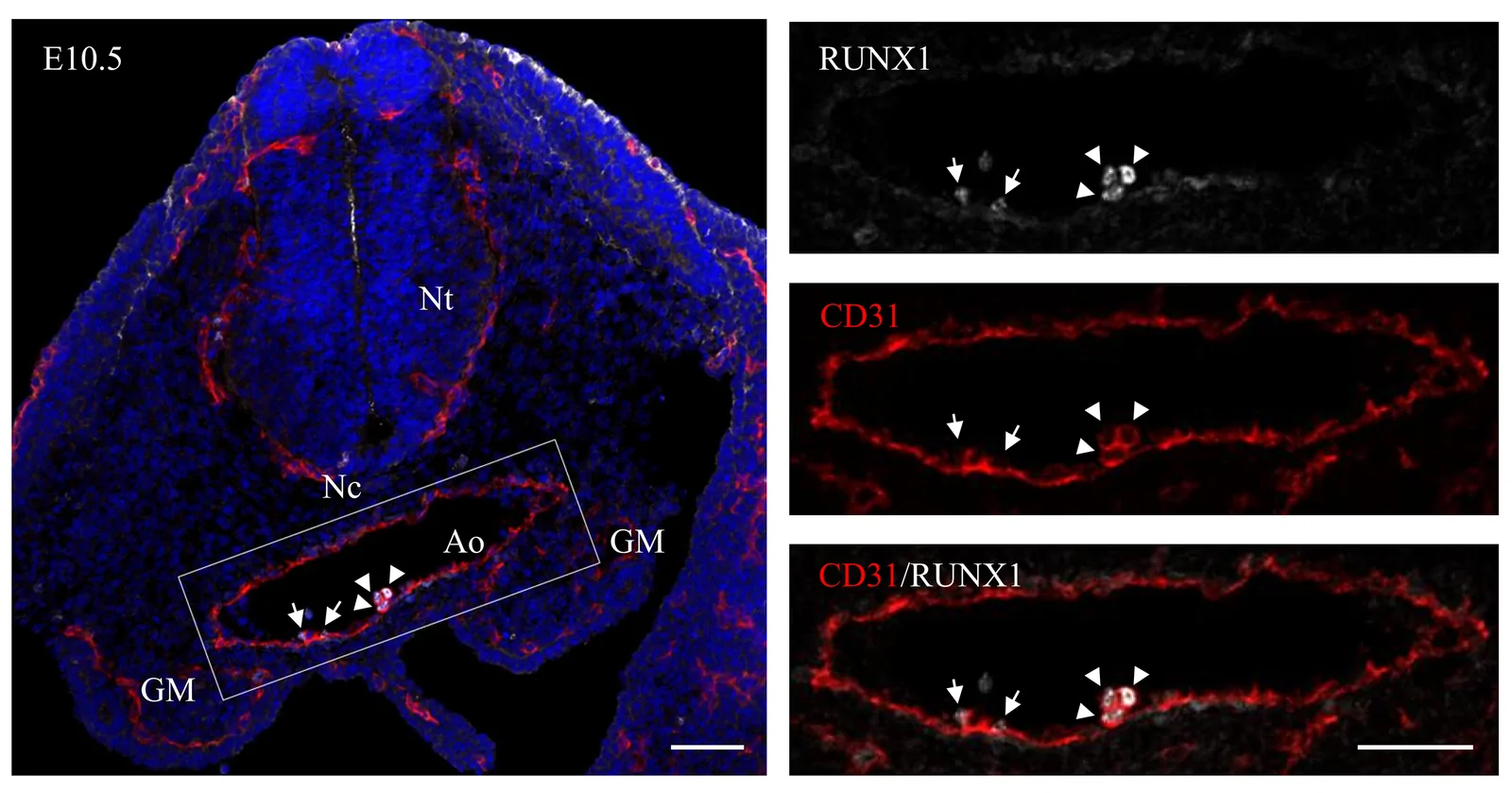
Representative immunofluorescence images of the E10.5 mouse AGM region. The left panel shows a low-magnification overview of a transverse section from an E10.5 mouse embryo, with the aorta (Ao) region indicated by a dashed box. Higher-magnification images of this area are shown on the right panel, including the RUNX1, CD31, and merged channels. RUNX1^+^ cells undergoing budding are indicated by white arrows, and intra-aortic hematopoietic clusters (IAHCs) are indicated by white arrowheads. Nt, neural tube; Ao, aorta; G, gonad; M, mesonephros; Nc, notochord. Scale bars: 50 μm.

## Validation of protocol

Frozen-section immunofluorescence staining of the mouse AGM region has been used in several recent studies [10,11,15–17].

However, none of the above studies provides a detailed step-by-step guide for precisely localizing the AGM region—a critical step that greatly affects experimental success. To fill this gap, we present an optimized, reproducible workflow that clearly describes embryo embedding orientation and rapid AGM region identification based on anatomical landmarks during cryosectioning. The protocol also includes immunofluorescence and confocal imaging procedures, offering a complete and fully reproducible experimental pipeline.

A manuscript employing this complete protocol is currently in preparation and is expected to be submitted within three to six months. Importantly, we have independently validated this protocol in our laboratory through multiple experimental replicates, consistently obtaining robust and reproducible results (representative data shown in Figure 5). These validations demonstrate that this protocol enables precise localization of the mouse embryonic AGM region and ensures clear signal detection without overexposure or photobleaching. Furthermore, the protocol has been successfully validated using additional antibodies beyond CD31 and RUNX1 (e.g., CD44, KIT, GFI1; Figure S2) and at different embryonic stages (E9.5 and E11.5; Figure S3). In all cases, following the described procedure yielded specific staining with high signal-to-noise ratios and accurate spatial localization.

## General notes and troubleshooting

**General notes**

1. Embryonic stage should be determined using both vaginal plug detection and somite counting, rather than relying on mating time alone. This is particularly critical when comparing hematopoietic development among embryos from different experimental groups.

2. For detection of endogenous fluorescent protein expression, sections should be observed as soon as possible after sectioning, ideally within three days. If endogenous fluorescence is not to be detected, sections can be stored at -80 °C and subjected to immunofluorescence staining within 1–2 weeks.

3. Embryonic cryosections are fragile and prone to detachment during immunofluorescence washing steps. The use of adhesion slides, gentle liquid handling, and low-speed shaking is recommended throughout the staining procedure to preserve tissue integrity.

4. Tissue drying is a common cause of high background and uneven staining in immunofluorescence. Therefore, sections should remain covered with solution during blocking, antibody incubation, and washing steps.

5. For quantitative analysis, all samples should be processed under identical conditions, including fixation time, antibody concentration, and imaging settings.

6. This protocol is designed for visualization and quantitative analysis of hematopoietic cells in the AGM region. It does not provide functional evidence of hematopoietic activity, which requires additional assays (e.g., colony-forming or transplantation assays).

**Troubleshooting**

**Problem 1:** Difficulty in orienting the embryo correctly during embedding.

Possible cause: The fluidity of O.C.T. compound allows the embryo to maintain an upright orientation for only a short time.

Solution: Prepare liquid nitrogen in advance. Once the embryo is positioned, immediately freeze it rapidly in liquid nitrogen.

**Problem 2:** Tissue cracking during cryosectioning.

Possible cause 1: Embryos may be over-dehydrated during sucrose cryoprotection.

Possible cause 2: Excessive residual moisture around the tissue during embedding.

Solution 1: For E10.5 embryos, incubate embryos in 15% sucrose/PBS at 4 °C for approximately 3 h. Do not exceed 4 h.

Solution 2: Use a lint-free wipe to absorb excess moisture around the tissue, but avoid drying the tissue.

**Problem 3:** Tissue dries during immunofluorescence staining.

Possible cause 1: The volume of the blocking solution or antibody solution may be insufficient.

Possible cause 2: The hydrophobic barrier may leak.

Possible cause 3: Slides may be exposed to air for too long.

Solution 1: Add enough solution to fully cover the tissue, usually 30–50 μL per section depending on tissue size.

Solution 2: Draw a complete hydrophobic barrier around each section, which must be done when the area around the tissue is completely dry.

Solution 3: Place slides in a humidified chamber during incubation.

**Problem 4:** High background fluorescence.

Possible cause 1: Excessive antibody concentration or incomplete washing.

Possible cause 2: Tissue drying during staining.

Solution 1: Optimize primary and secondary antibody dilutions before the formal experiment. Wash slides thoroughly with PBS at 20 rpm, keeping all tissues immersed in liquid during washing.

Solution 2: Keep sections wet throughout staining.

**Problem 5:** Weak or absent fluorescence signal.

Possible cause 1: Antibody concentration is too low, or the antibody quality is poor.

Possible cause 2: Samples are exposed to light for too long.

Possible cause 3: Confocal imaging settings are inappropriate.

Solution 1: Increase the antibody concentration or replace the antibody.

Solution 2: Protect slides from light after secondary antibody incubation.

Solution 3: Appropriately increase laser power or detector gain while avoiding overexposure.

## Supplementary information

The following supporting information can be downloaded here:

1. Figure S1. Representative cryosection lacking the correct anatomical location and characteristic landmarks of the AGM region.

2. Figure S2. Immunofluorescence of GFI1, KIT, and CD44 in the E10.5 mouse AGM region.

3. Figure S3. Immunofluorescence of CD31 in the mouse AGM region at different stages.
